# Are some feasibility studies more feasible than others? A review of the outcomes of feasibility studies on the ISRCTN registry

**DOI:** 10.1186/s40814-021-00931-y

**Published:** 2021-11-08

**Authors:** Ben Morgan, Jennie Hejdenberg, Kasia Kuleszewicz, David Armstrong, Sue Ziebland

**Affiliations:** 1 National Institute for Health Research Central Commissioning Facility, Twickenham, UK; 2 Department of Primary Care & Public Health Sciences, King’s College London, London, UK; 3 Nuffield Department of Primary Care Health Sciences, University of Oxford, Oxford, UK

**Keywords:** Feasibility studies, Research waste, Progression rates

## Abstract

**Background:**

Feasibility studies are often conducted before committing to a randomised controlled trial (RCT), yet there is little published evidence to inform how useful feasibility studies are, especially in terms of adding or reducing waste in research. This study attempted to examine how many feasibility studies demonstrated that the full trial was feasible and whether some feasibility studies were inherently likely to be feasible or not feasible, based on the topic area and/or research setting.

**Methods:**

Keyword searches were conducted on the International Standard Randomised Controlled Trials Number (ISRCTN) registry to identify all completed feasibility studies which had been conducted in the UK.

**Results:**

A total of 625 records from the 1933 identified were reviewed before it became evident that it would be futile to continue. Of 329 feasibility studies identified, 160 had a known outcome (49%), 133 (83%) trials were deemed to be feasible and only 27 (17%) were reported to be non-feasible. There were therefore too few studies to allow the intended comparison of differences in non-feasible studies by topic and/or setting.

**Conclusions:**

There were too few studies reported as non-feasible to draw any useful conclusions on whether topic and/or setting had an effect. However, the high feasibility rate (83%) may suggest that non-feasible studies are subject to publication bias or that many feasible studies are redundant and may be adding waste to the research pathway.

## Background

Large randomised controlled trials (RCTs) are expensive and time-consuming and often require substantial and sustained commitment from patients and clinical staff. It has become increasingly common to conduct preliminary studies, often using a pilot or feasibility design, to mitigate the risks and financial commitment involved in running a multicentre RCT. As demonstrated by Chalmers and Glasziou, a substantial amount of research waste occurs when inappropriate questions are asked, inappropriate methods used and the results are inappropriately reported or not made available [1, 2]. Getting this right in large clinical trials matters and preliminary, feasibility studies ought to help. In this study, we used the definition by Eldridge et al. [3], which considers pilot studies as a subset of feasibility studies in preparation for a future RCT.

There have been very few studies examining whether feasibility studies really do succeed in mitigating risks and improving trial design, rather than simply adding another layer of time and expense to the research pathway. One such example, Charlesworth et al. showed that only 53.8% of correctly labelled pilot studies reported in six anaesthesia journals had progressed into a subsequent study 5 (or more) years later [4].

Research examining the conduct of feasibility studies has concluded that these are often suboptimal and has proposed recommendations to improve how the studies should be described, conducted and reported. Contributions include the conceptual framework developed by Eldridge et al. [3], Thabane & Lancaster’s guide to the reporting of protocols of pilot and feasibility trials [5], the CONSORT extension checklist for reporting pilot studies [6] and a specific website for pilot and feasibility studies which hosts various resources to aid researchers in the design, analysis and reporting of studies [7].

Whilst these contributions [4–7] take an overarching perspective on the design and conduct of feasibility studies, a recent trend has focussed on their role and effectiveness in specific disciplines or in particular trial designs. Kosa et al. found that pilot studies in chronic kidney disease patients on haemodialysis did not typically adhere to CONSORT reporting guidelines; however, improvements were noted in the reports of larger and more recent studies [8]. Fairhurst et al. concluded that pilot and feasibility studies conducted in surgery fail to fully address the uncertainties specifically related to surgical RCTs and instead focus on generic uncertainties [9]. Charlesworth et al.’s review of six anaesthesia journals found that only 12.8% of the studies described as ‘pilot studies’ met the agreed criteria. They concluded that better reporting in line with CONSORT was needed [4]. Relating more to methods than discipline, Rosala-Hallas et al. reviewed internal pilots of the National Institute for Health Research (NIHR) Health Technology Assessment (HTA) programme and showed that not all had clear progression criteria, and in some cases, studies would progress to a full RCT without the criteria being met [10]. Kristunas et al. reviewed the role of feasibility studies in stepped-wedge cluster randomised trials. Few relevant studies were found and most of these did not examine the specific challenges associated with conducting stepped-wedge cluster randomised trials [11].

Whilst these reports address the quality of feasibility studies, few studies have addressed whether feasibility studies can mitigate risk and improve trial design. Previous analysis of feasibility studies funded by the National Institute for Health Research’s (NIHR) Research for Patient Benefit’s (RfPB) programme showed that on average a feasibility study demonstrated that the RCT was feasible in 64% of cases [12]. The average cost of a feasibility study was £219,048 and took 31 months to complete. A typical RCT following an RfPB feasibility study costs on average £1,163,996 and took 42 months to complete. Thus, a significant amount of time and funding is spent on feasibility studies: is this a good use of resources?

Whilst there are clear reporting guidelines for pilot and feasibility studies and materials to help researchers conduct these studies more appropriately, the available evidence suggests many pilot and feasibility studies are still poorly conducted and reported. It seems likely therefore that these studies are not currently reaching their full potential to help reduce waste in research.

Researchers and research funders have more to learn about whether and how pilot and feasibility studies add value. There may, for example, be nuances related to particular disciplines or methods. When reviewing the RfPB feasibility studies [12], it was noted that although the overall feasibility rate was 64%, it appeared to vary by research topic and settings. The RfPB data did not include sufficient studies to answer this question with confidence, yet, given the time and expense involved in RCTs, it is worth exploring whether feasibility studies in certain topics or settings are more likely to indicate that an RCT is feasible.

To answer this question, we examined feasibility studies registered on the International Standard Randomised Controlled Trials Number (ISRCTN) registry. If we could establish that feasibility studies in certain topic areas or settings were more or less feasible than the RfPB average of 64%, it could provide researchers and funders with better information before committing to a particular feasibility study. For example, if some feasibility studies in certain topics/settings had a high chance of demonstrating feasibility, it may be more appropriate to go straight to RCT as there is less risk involved. Alternatively, if we discovered that feasibility studies in certain topic areas or settings often indicated that an RCT would not be feasible—and we could determine the reasons why—it could help researchers to design more appropriate studies. We therefore set about examining the extent to which feasibility studies reported in a large trials database (ISRCTN) found a potential trial was ‘not feasible’ and whether this latter finding varied for clinical topics and settings.

## Methods

A keyword search was conducted on 03 March 2019 on the ISRCTN database using the search terms ‘pilot’, ‘feasibility’ and ‘feasible’. Data were extracted in Microsoft Excel. Duplicate entries were removed. The unique list of records was filtered by ‘Country of recruitment’ as ‘UK’ and ‘Overall trial status’ as ‘Complete’. ‘Condition Category’ was used to determine the research topic area and ‘Trial Setting’ was used to determine the research setting. ‘Condition Category’ is selected by ISRCTN editorial staff and is based on International Classification of Diseases (ICD) – 10. ‘Trial Setting’ is a self-reported option researchers select from a dropdown list [13].

Using Eldridge et al.’s [3] view of pilot and feasibility studies as preparatory studies for a future RCT, with pilot studies as a subset of feasibility studies, the individual records were reviewed independently by two authors to determine:

If the research was a feasibility study (i.e. explicitly stated it was preparing for a RCT and had typical feasibility outcomes (for clarity studies described as pilot studies were included if explicitly stated to be in preparation for a RCT).

Findings and interpretations reported by the researchers in their results papers were then used to determine:

1. If the research was a feasibility study, did it demonstrate the RCT to be feasible?
2. If it was a feasibility study and the RCT was not feasible, the reasons why it was not feasible

Specific reasons given for why a RCT was not feasible (item 3) were themed into:

- Patient recruitment—where difficulties were encountered in recruiting and/or retaining the participants during the study or low eligibility of patients
- Trial design/methods—where difficulties were encountered in blinding, inappropriate inclusion/exclusion criteria, general method/design issues
- Intervention—where the intervention lacked sufficient promise of efficacy/effectiveness, was not used by participants, or was not practical
- Outcome measures—where outcome measures were not acceptable or could not be collected.

Where multiple reasons why a RCT was not feasible were reported, these were counted separately, i.e. a single feasibility study could have more than one reason why the RCT was not feasible.

The inter-rater reliability was calculated using percentage agreement between raters. Where there was disagreement between the two reviewers on points 1, 2 or 3, a third reviewer adjudicated.

The sources of information to answer points 1, 2 or 3 were:

- The content on the ISCTRN record
- Links to published papers from the ISCTRN record
- Where no links to published papers were noted in the ISCTRN records, a search was conducted on Europe PMC to see if any published papers could be found. Only papers with a clear link to the research were included, e.g. ISRCTN number referenced in the paper.

Where there were multiple sources of information available for a single study, such as the ISRCTN record, a protocol paper and results paper we used the latest available information, for example, results papers were used over protocol papers, and protocol papers were used over the ISRCTN records, etc.

## Results

A total of 1933 unique study entries were returned from the keyword searches once filters had been applied to ‘Country of recruitment’ as ‘UK’ and ‘Overall trial status’ as ‘Complete’. Whilst reviewing the records, it became clear there would be an insufficient number of studies where the RCT was considered *not* feasible to make a credible assessment of whether research topic and/or research setting were related to conclusions about feasibility. Having found only 27 non-feasible studies from an initially randomly selected pool of 625 records, we estimated that the whole dataset of 1933 records would be unlikely to yield more than 84 non-feasible studies, an insufficient number to evaluate the effect of clinical topic or setting. Therefore, only 625 records were included in the analysis below. The 625 studies had trial end dates between 1995 and 2019 with the distribution shown in Table 1. From the 625 records reviewed, only 329 (53%) were agreed to be feasibility studies, using the Eldridge definition (3).

**Table 1:** Distribution of ISRCTN records by trial end date

Trial end date	Total sample (***n*** = 1933) ***n*** (%)	Reviewed sample (***n*** = 625) ***n*** (%)	Number RCT not feasible (***n*** = 27) ***n*** (%)
1993	1 (0.1)	0 (0)	0 (0)
1994	0 (0)	0 (0)	0 (0)
1995	1 (0.1)	1 (0.2)	0 (0)
1996	3 (0.2)	0 (0)	0 (0)
1997	7 (0.4)	1 (0.2)	0 (0)
1998	5 (0.3)	2 (0.3)	0 (0)
1999	9 (0.5)	2 (0.3)	0 (0)
2000	9 (0.5)	3 (0.5)	0 (0)
2001	14 (0.7)	4 (0.6)	0 (0)
2002	14 (0.7)	1 (0.2)	0 (0)
2003	51 (2.6)	11 (1.8)	0 (0)
2004	50 (2.6)	14 (2.2)	0 (0)
2005	50 (2.6)	14 (2.2)	0 (0)
2006	86 (4.4)	19 (3.0)	0 (0)
2007	88 (4.6)	22 (3.5)	0 (0)
2008	64 (3.3)	21 (3.4)	0 (0)
2009	64 (3.3)	19 (3.0)	2 (7.4)
2010	88 (4.6)	20 (3.2)	1 (3.7)
2011	110 (5.7)	33 (5.3)	2 (7.4)
2012	131 (6.8)	32 (5.1)	1 (3.7)
2013	133 (6.9)	37 (5.9)	1 (3.7)
2014	169 (8.7)	52 (8.3)	7 (25.9)
2015	144 (7.4)	46 (7.4)	3 (11.1)
2016	161 (8.3)	61 (9.8)	3 (11.1)
2017	201 (10.4)	85 (13.6)	5 (18.5)
2018	210 (10.9)	97 (15.5)	2 (7.4)
2019	70 (3.6)	28 (4.5)	0 (0)

Of the 329 identified as feasibility studies:

- 133 studies (40%) indicated that the RCT was feasible
- 27 studies (8%) indicated that the RCT was not feasible
- For 160 studies (49%), the outcome was unknown because no results could be found
- 97 studies (61%) of these 160 studies were within 24 months of ‘Overall trial end’ while 63 studies (39%) had not published results between two and twenty years since the ‘Overall trial end’
- In 8 studies (2%), it was unclear if they were feasibility studies or not due to insufficient reported detail
- 1 study terminated before it started

Therefore, from the 160 records with known feasibility outcomes, 133 (83%) concluded that the RCT was feasible and 27 (17%) that the RCT was not feasible. The interrater reliability across the three rating points was 84%.

The breakdown of the results by topic and setting are presented in Tables 2 and 3, albeit based on small numbers in Table 2, where there were at least 10 studies reported for a specific research topic, the feasibility rate ranged between 79% and 100% for individual research topics, and in Table 3, the feasibility rate ranged between 73% and 93% for research setting.

**Table 2:** Breakdown of research topic area and whether RCT was feasible or not feasible for studies with known outcomes

Research topic	Total (***N*** = 160) ***n*** (% of total)	RCT feasible (***n*** = 133) ***n*** (% of topic)	RCT not feasible (***n*** = 27) ***n*** (% of topic)
Cancer	19 (12)	15 (79)	4 (21)
Circulatory System	19 (12)	19 (100)	0 (0)
Digestive System	4 (3)	3 (75)	1 (25)
Ear, Nose and Throat	0 (0)	0 (0)	0 (0)
Eye Diseases	2 (1)	2 (100)	0 (0)
Genetic Diseases	0 (0)	0 (0)	0 (0)
Infections and Infestations	4 (3)	1 (25)	3 (75)
Injury, Occupational Diseases, Poisoning	2 (1)	0 (0)	2 (100)
Mental and Behavioural Disorders	39 (24)	33 (85)	6 (15)
Musculoskeletal Diseases	7 (4)	5 (71)	2 (29)
Neonatal Diseases	1 (1)	1 (100)	0 (0)
Nervous System Diseases	9 (6)	8 (89)	1 (11)
Not Applicable	8 (5)	5 (63)	3 (38)
Nutritional, Metabolic, Endocrine	15 (9)	13 (87)	2 (13)
Oral Health	1 (1)	1 (100)	0 (0)
Pregnancy and Childbirth	5 (3)	5 (100)	0 (0)
Respiratory	4 (3)	3 (75)	1 (25)
Signs and Symptoms	10 (6)	9 (90)	1 (10)
Skin and Connective Tissue Diseases	1 (1)	1 (100)	0 (0)
Surgery	6 (4)	5 (83)	1 (17)
Urological and Genital Diseases	4 (3)	4 (100)	0 (0)

**Table 3:** Breakdown of research setting and whether RCT was feasible or not feasible for studies with known outcomes

Research Setting	Total (***N*** = 160) ***n*** (% of total)	RCT feasible (***n*** = 133) ***n*** (% of setting)	RCT not feasible (***n*** = 27) ***n*** (% of setting)
Community	3 (2)	2 (67)	1 (33)
GP practices	15 (9)	11 (73)	4 (27)
Home	1 (1)	1 (100)	0 (0)
Hospitals	80 (50)	69 (86)	11 (14)
Internet	0 (0)	0 (0)	0 (0)
Not specified	14 (9)	13 (93)	1 (7)
Other	37 (23)	30 (81)	7 (19)
Schools	10 (6)	7 (70)	3 (30)

**Fig. 1:**
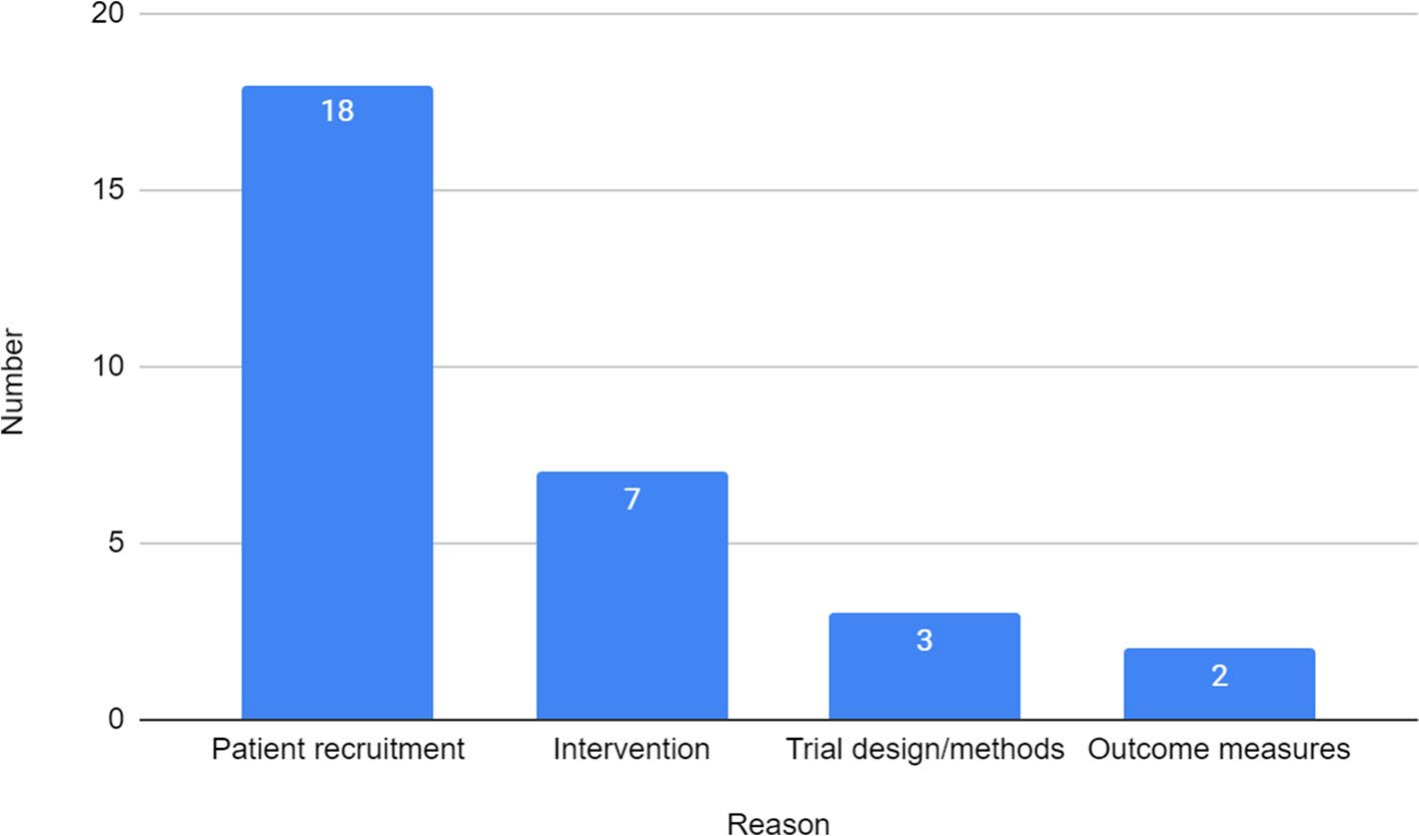
Reasons why RCTs were not feasible

Figure 1 shows the specific reasons RCTs were deemed not to be feasible, irrespective of research topic area or research setting, with patient recruitment clearly being the most common reason and reported for 18 (60%) of the 27 feasibility studies reporting the RCT to be not feasible.

The largest outcome group was ‘unknown if RCT feasible’ (*n* = 160) which was where the feasibility study had completed but the published results could not be found. The trial end date of these studies ranged from 1999 to 2019. Sixty three of the 160 studies had trial end dates at least 2 years before our data access and therefore would have had ample time to publish. This suggests that there is a large amount of under-reporting of results of feasibility studies which is consistent with the general under-reporting of trials [14–18].

## Discussion

It was not possible to determine whether certain research topics and/or research settings are more associated with feasibility because we found too few studies reporting that the RCT was not feasible. Irrespective of research topic or research setting, there were consistent reasons why RCTs were considered not feasible: patient recruitment, trial design/methods, intervention and outcome measures. Of these, patient recruitment was the most commonly reported reason in the published results paper. This will not be a controversial finding for the research and funding community.

There are several plausible reasons why studies showing that RCTs are not feasible do not often appear in the literature. First, whilst feasibility studies are typically smaller than RCTs, it is clear from RfPB data [12] that feasibility studies are still a large investment of resource costing on average £219,048 and taking 31 months. Research teams may be confident (and persuasive) enough to apply for the full RCT, perhaps including an internal pilot. Second, it is becoming more routine to include feasibility progression criteria in feasibility designs with ‘red, amber, green’ pre-specified assessments to indicate feasibility. Many of the studies we reviewed did not use progression criteria or used unclear criteria. Therefore, it is very plausible that some studies were optimistic in reporting the RCT to be feasible. We also noted examples where researchers had reported the RCT feasible subject to such substantial changes that it was debatable what relation the feasibility study could claim to any eventual RCT. Third, the bar to demonstrate feasibility may be artificially low if studies are not identifying and addressing the specific challenges in the clinical field of interest. This may be accentuated if feasibility studies rely on a formulaic design [8]. Fourth, the team may be reluctant to publish the conclusion that a trial is not feasible. Historically, there have been challenges publishing ‘negative results’, but recent advances such as better reporting guidance (CONSORT) and specialist journals such as *Pilot and Feasibility Studies* have helped to address these challenges. Looking at the 160 studies that apparently had no results published, 63 had finished more than 2 years before our data access and thus would have had ample time to publish. The remaining 97 studies may well go on to publish non-feasible conclusions in future. Therefore, future reviews of feasibility studies might show a more complete publication picture.

There were some challenges in reviewing the ISRCTN records. Firstly, although we used a clear definition of what constitutes a feasibility study, to some extent, we were retro-fitting a definition to previously funded research. Nevertheless, we took the view that any study that explicitly stated it was preparing for a RCT, and had typical feasibility outcomes, was eligible for inclusion. One particular challenge was the number of studies that identified as pilot studies yet had no explicit plan to conduct a further RCT. Some included a mix of typical feasibility study outcomes and typical pilot study outcomes. These were typically excluded if they were primarily exploratory studies or even underpowered trials. Another challenge from the ISRCTN records was that we found self-identified feasibility studies which were examining the feasibility of implementation of a particular treatment/service rather than examining whether a future RCT was feasible. Studies that were not planning to progress to a RCT were excluded but were often challenging to identify.

It is clear from the data that more feasibility studies, or at least more studies which resemble current definitions of feasibility studies, are being conducted, with over 97 feasibility studies completing in 2018 compared with 21 10 years earlier and two 10 years before that. This follows the trend of more focus on feasibility studies with activity such as more research, specialised journals and better guidance on designing, conducting and reporting feasibility studies. It would be interesting to understand why more feasibility studies are being conducted. Could it be that funders of RCTs require more evidence that expensive RCTs will be successfully delivered? Perhaps there are also more funding opportunities for feasibility studies with NIHR programmes like RfPB becoming well known for supporting such studies. It is likely that more feasibility studies are now included on trial registries and were therefore discoverable.

Whilst the majority of the feasibility studies with reported outcomes are feasible, it would have been useful to have known how many studies progressed to RCT and how long they took to progress to have a better-informed view of the utility of feasibility studies. Whilst we noticed that some ISRCTN records contained both the feasibility study and subsequent RCT in a single ISRCTN record, it was not clear how systematically these records were updated. It would be useful if trial registries collected more data including links between feasibility studies and RCTs and ultimately outcomes from RCTs. Knowing the full picture from feasibility study progression to RCT competition and study outcome would allow a more informed view on how feasibility studies contribute to the trials pathway.

It is reasonable to assume that if the feasibility rate is too high, then researchers and research funders are contributing to the waste in the system by conducting studies which will often show the RCT is feasible. In such cases, it may be more appropriate to go straight to the RCT and accept that a relatively small percent will inevitably fail, whilst noting that feasibility studies do help address other uncertainties which may not have been foreseen. Conversely, if the feasibility rate is too low, it may indicate that researchers and funders are too optimistic and commit to studies which are not likely to progress down the trials pathway, although based on the current analysis, there is insufficient evidence to suggest that this scenario is occurring. However, it is acknowledged that many useful aspects and insights are gained via feasibility studies and that those demonstrating the RCT to be feasible often do once addressing challenges encountered via the feasibility study.

There must be a point where if the feasibility rate is too high it would be more cost and time effective to fund studies as RCTs and accept that a certain percentage will inevitably fail. However, for those that do fail, a greater amount of time and/or funding will have been saved by successfully completed RCTs which avoided the feasibility and/or pilot step. The cost of the ISRCTN feasibility studies was not reported, but based on the previous RfPB review [12], the average feasibility study cost £219,048 and took 31 months. If the 83% feasibility rate of ISRCTN studies is correct, then that is a substantial amount of funding and time taken up by the feasibility studies when only approximately 1 in 5 will show the RCT to not be feasible. Using the average cost of feasibility studies (£219,048) and RCTs (£1,163,996) from our previous review of RfPB studies [12], we can begin to estimate what an appropriate feasibility rate may be. RfPB feasibility studies were shown to be feasible in approximately two out of three studies, and therefore approximately £657,144 was spent on feasibility studies to save up to approximately £1,163,996 for the RCT which was not feasible. If we apply the same estimates based on the ISRCTN feasibility rate of 83% (approximately four out of five studies), then approximately £1,095,240 needs to be spent on feasibility studies to save up to approximately £1,163,996 for the RCT which was not feasible. In addition to cost, there is also a time addition as each feasibility study takes an average 31 months to complete. A feasibility rate of 83% appears too high and would suggest feasibility studies are wasteful, whereas 64% may be considered more reasonable.

How many of these RCTs genuinely needed a feasibility study and then focussed on the actual uncertainties instead of adopting a generic design? Perhaps even more important is how many of those 83% which demonstrate feasibility actually progress to RCT? Feasibility studies which show the RCT feasible but which do not progress to RCT, for various reasons, could also be considered to be adding to research waste, especially if these feasibility studies are not really needed in the first place. The answer to this question is made challenging by historic poor reporting and publication rates of feasibility studies.

This study raises, but cannot address, the question of what would be an acceptable ‘success’ rate for feasibility studies: should most demonstrate feasibility of the RCT or should more demonstrate the RCT not feasible? How much risk do funders and researchers want to take? Might shorter and more cost effective feasibility studies be more informative? Perhaps the view that feasibility studies are essential before conducting a RCT is leading to the design of studies which are likely to ‘succeed’ and therefore lack equipoise or do not focus on the most important uncertainties which need to be addressed in relation to the specific trial. As shown by the existing literature, it is often the case that feasibility studies in certain topic areas do not maximise their potential benefit and focus on the key uncertainties [8, 11] and instead adopt a generic design.

Although we were unable to answer our initial question, a potentially more important and interesting question is what the rate of feasibility studies demonstrating the RCT is feasible should be. The previous review of the RfPB portfolio [12] showed that 64% of studies demonstrated the RCT to be feasible and this review of ISRCTN registered studies showed that 83% of studies with known/published outcomes demonstrated the RCT feasible. Are these feasibility rates appropriate and what do they mean for the wider trajectory along the trials pathway?

## Conclusion

It is likely that there are insufficient published studies demonstrating that the RCT is not feasible to be able to assess whether some studies may or may not be more feasible than average based on research topic and/or research setting. More discussion is required between researchers, methodologists and research funders on exactly what feasibility studies are aiming to achieve and what proportion of studies should demonstrate feasibility or not and how this relates to the wider research, funder and patient benefit pathway. This will help ensure that feasibility studies maximise the potential to reduce waste in research instead of potentially adding to it.

## Limitations

The sample included a range of studies spanning over 20 years. During this time, research design and conduct has changed including definitions of ‘feasibility study’. To that end, we applied a relatively recent definition of ‘feasibility study’ to historical studies which made reviewing studies challenging. The potential under-reporting of non-feasible studies possibly biased the sample leading to an artificially high feasibility rate.

## Data Availability

All data generated or analysed during this study are included in this published article and its supplementary information files.

## References

[1] Chalmers I, Glasziou P (2009). Avoidable waste in the production and reporting of research evidence. Lancet.

[2] Chalmers I, Bracken MB, Djulbegovic B, Garattini S, Grant J, Gülmezoglu AM (2014). How to increase value and reduce waste when research priorities are set. Lancet.

[3] Eldridge SM, Lancaster GA, Campbell MJ, Thabane L, Hopewell S, Coleman CL (2016). Defining feasibility and pilot studies in preparation for randomised controlled trials: development of a conceptual framework. PLoS One.

[4] Charlesworth M, Klein AA, White SM (2020). A bibliometric analysis of the conversion and reporting of pilot studies published in six anaesthesia journals. Anaesthesia.

[5] Thabane L, Lancaster G (2019). A guide to the reporting of protocols of pilot and feasibility trials. Pilot Feasibility Stud.

[6] Eldridge SM, Chan CL, Campbell MJ, Bond CM, Hopewell S, Thabane L (2016). CONSORT 2010 statement: extension to randomised pilot and feasibility trials. BMJ.

[7] Chan CL (2019). A website for pilot and feasibility studies: giving your research the best chance of success. Pilot Feasibility Stud.

[8] Kosa SD, Monize J, Leenus A (2019). Reporting quality of pilot clinical trials in chronic kidney disease patients on hemodialysis: a methodological survey. Pilot Feasibility Stud.

[9] Fairhurst K, Blazeby JM, Potter S, Gamble C, Rowlands C, Avery KNL (2019). Value of surgical pilot and feasibility study protocols. Br J Surg.

[10] Rosala-Hallas A, Gamble C, Blazeby J (2019). A review of current practice in the design and assessment of internal pilots in UK NIHR clinical trials. Trials.

[11] Kristunas CA, Hemming K, Eborall H (2019). The current use of feasibility studies in the assessment of feasibility for stepped-wedge cluster randomised trials: a systematic review. BMC Med Res Methodol.

[12] Morgan B, Hejdenberg J, Hinrichs-Krapels S, Armstrong D (2018). Do feasibility studies contribute to, or avoid, waste in research?. PLoS One.

[13] ISRCTN registry: Definitions, http://www.isrctn.com/page/definitions [Date accessed: 03 Mar 2019]

[14] DeVito NJ, Bacon S, Goldacre B. Compliance with legal requirement to report clinical trial results on ClinicalTrials.Gov: a cohort study. Lancet. 2020published online Jan 17. 10.1016/S0140-6736(19)33220-9. 10.1016/S0140-6736(19)33220-9 31958402

[15] Dwan K, Gamble C, Williamson PR, Kirkham JJ, the Reporting Bias Group (2013). Systematic review of the empirical evidence of study publication bias and outcome reporting bias — an updated review. PLoS One.

[16] Schmucker C, Schell LK, Portalupi S, Oeller P, Cabrera L, Bassler D (2014). Extent of non-publication in cohorts of studies approved by research ethics committees or included in trial registries. PLoS One.

[17] Goldacre B, DeVito N J, Heneghan C, Irving F, Bacon S, Fleminger J, et al. Compliance with requirement to report results on the EU Clinical Trials Register: cohort study and web resource. BMJ. 2018;362:k3218. 10.1136/bmj.k3218. 10.1136/bmj.k3218 PMC6134801 30209058

[18] Anderson ML, Chiswell K, Peterson ED (2015). Compliance with results reporting at ClinicalTrials.Gov. N Engl J Med.

